# Book Reviews

**Published:** 1874-06-15

**Authors:** 


### Book Reviews.

LIGATION of Arteries. An operative manual by Dr. L. H. Farrabeuf, Aide d'Anatomie a la Faculte, Ancien Interne des Hopitaux de Paris. Translated by John D. Jackson, M.D., of Danville, Kentucky. With engravings; pp. 157. Philadelphia: J. B. Lippincott & Co., 1874.

The unpretentiousness of this little volume is no measure of its excellence. The author desires to inform the would-be operator, not only as to what is to be done and what is to be left undone, but precisely *how* to do the former. After carefully reviewing the treatises on operative surgery, published in his own country and abroad, he proceeds to produce a work which does not presume to describe all known processes for artery ligation, but those preferred by most eminent surgeons. In this respect, it is an exceedingly valuable and compendious treatise.

To Dr. J. D. Jackson, we beg leave to extend our thanks and congratulations. He has demonstrated that some good can come out of Nazareth.

From the host of illy-devised, slovenly-written, poorly-translated works of Western and Southern medical men, we select his little volume as an evidence of the better things of which they are capable. It is fairly in an English dress, and does not wear the garments of a harlequin or a distinguished foreigner. There is nothing careless or slip-shod in his version. It is, in short, well done. The volume is elegantly produced by Messrs. Lippincott & Co., and the forty-three plates are faultlessly executed. "*Rari nantes in gurgite vasto.*"

Lectures on the Diseases of Infancy and Childhood. By Charles West, M. D., Fellow of the Royal College of Physicians; Physician to the Hospital for Sick Children; 5th American; from the 6th Revised and Enlarged English Edition. Philadelphia: Henry C. Lea, 1874; pp. 663. Chicago: Jansen, McClurg & Co.

This work has been so long and so favorably known to the medical profession everywhere, that another edition calls for little more than a word of welcome. Praise is superfluous where merit has already gained its deserved reward. The perfect clearness of the style of the author, the practical character of his instruction, and the completeness of the study of each special disease, render the volume indispensable to every practitioner who is called upon to treat children.

Dr. West is now sixty years old, and can refer to a period of thirty years past, when there was not a single hospital for children in Britain or America. If we have to-day institutions of that character, reared by a large-handed liberality, and conducted in accordance with a method whose progress has almost created a new field of medical science, we owe it to Charles West, and his co-laborers, in the department of Diseases of Children.

A Treatise on Pharmacy. Designed as a text-book for the Student, and as a guide for the Physician and Pharmacist, &c., &c. By Edward Parrish. Fourth edition, enlarged and revised by Thos. S. Wiegand. Chicago: W. B. Keen, Cooke & Co.

Parrish's Practical Pharmacy has long held a high rank amongst works on Pharmaceutical Science. Physician and Pharmacist have alike regarded it as the standard on all subjects of which it treated. It is with pleasure, therefore, that we welcome a new edition of this excellent work. After the sudden death of Prof. Parrish, in the fall of 1872, the question was often asked: How about the new edition of his work? on the preparation of which he was known to be engaged. Considerable uncertainty existed on the subject, till it was known that Mr. Thos. S. Wiegand, of Philadelphia, had undertaken the onerous duty of completing the work begun by Mr. Parrish. That he has carefully and faithfully performed this difficult and arduous task, the volume before us affords ample proof. During the decade which has passed since the last edition was published, many new discoveries have been made in chemical science; many old theories have been exploded, and many new ones advanced. The United States Pharmacopœia has been revised, changing many old formulæ, and adding numerous new ones. The new notation has also been almost universally adopted. All these points have been duly taken into consideration by Mr. Wiegand. The latter, that of figuring out all chemical formulæ, in accordance with the new notation, must have been of itself no mean undertaking.

The work is divided into six parts, with an appendix, and, although considerably changed in arrangement, the general design of the former editions has been retained. With these few remarks we heartily commend the work, and have no doubt but it will maintain its old reputation, as a text-book for the student and a work of reference for the more experienced physician and pharmacist.

---

THE INFLUENCE OF NITROGENOUS MATTER UPON THE SECRETION OF GASTRIC JUICE.—At a recent meeting of the Biological Society of Paris [*Jour de Therap.*], an explanation was given, by M. Levin, regarding experiments tending to prove that the introduction of nitrogenous matter into the stomach brings to the gastric glands certain materials which promote the production of the gastric juice. Indeed, if two and a half ounces of some fatty substance, such as oil, for example, are placed in the stomach of a dog, upon killing the animal an hour subsequently, it will be found that the liquid in the stomach contains but a very slight amount of pepsin, and very often that it is entirely neutral to re-agents. So long as non-nitrogenous matter remains in the stomach, that organ secretes nothing but a liquid, which is absolutely destitute of gastric juice. It seems that the stomach has need of albuminoid aliments to facilitate its normal action, and to allow it to secrete upon its mucus surface a liquid of active reaction, and charged with pepsin. F. J. H.

---

THE leaves of the *eucalyptus globulus*, smoked in the form of cigarettes, are reported by the French medical journals to be efficacious in cases of bronchial and asthmatic affections.

